# A Redox Conjugated Polymer-Based All-Solid-State Reference Electrode

**DOI:** 10.3390/polym10111191

**Published:** 2018-10-25

**Authors:** Ke Qu, Mingxi Fang, Shuwei Zhang, Haiying Liu, Xiangqun Zeng

**Affiliations:** 1 Department of Chemistry, Oakland University, Rochester, MI 48309, USA; pdfgt25@163.com; 2 Department of Chemistry, Michigan Technological University, Houghton, MI 49931, USA; mfang@mtu.edu (M.F.); shuweiz@yzu.edu.cn (S.Z.); hyliu@mtu.edu (H.L.)

**Keywords:** redox conjugated polymer, polyaniline, electro-polymerization, quinone, reference electrode

## Abstract

This work reports the design, synthesis, and characterization of a novel redox-active conjugated polyaniline containing quinone moiety as a solid state reference electrode. The union of electro-active quinone with π-conjugated polyaniline was created by the first chemical synthesis of *para*-dimethoxybenzene-functionalized aniline as a monomer using a palladium-mediated coupling. The successful polymerization of the as-prepared monomer was accomplished without acid additives. Its post-polymerization modification with strong Lewis acid boron tribromide furnished unique poly (aniline quinone/hydroquinone) with desired properties for all-solid-state reference electrode (RE) applications. The electrochemical responses from the conjugated polyaniline backbone in this unique polymer have been “suppressed” by the quinone pendant. The resulting poly (aniline quinone) showed a quasi-reversible redox process from the redox behavior of the pendant quinone. The stable electrode potential of this poly (aniline quinone/hydroquinone) suggested that it was a single phase in which the amounts of totally reduced and totally oxidized species could be maintained at a constant in various solvents and electrolytes. Its electrochemical stability was excellent with 95% peak current retention after continuous cyclic voltammetric testing. The aniline and quinone moieties in poly (aniline quinone/hydroquinone) render it to have both hydrophilic and hydrophobic compatibility. It showed excellent behavior as a reference electrode in aqueous and non-aqueous media and can be used in both non-zero current and zero-current conditions, providing a stable potential with a maximum potential drift of ~4.7 mV over ten consecutive days.

## 1. Introduction

Electrochemistry is a discipline that studies chemical reactions that involve the exchange of electric charges between two substances. Electrochemistry has significant applications, ranging from batteries, sensors, corrosion control to electrolysis [1]. A reference electrode (RE) is a key component in electrochemistry research and applications. To monitor the charge transfer reaction at an electrode, a potential vs. RE is applied to the working electrode. A reference electrode is required to have a stable electrode potential, which is usually achieved by employing a redox system with constant (buffered or saturated) concentrations of each participant of the redox reaction [2]. Conventional REs include Ag/AgCl electrode and saturated calomel electrode (SCE), which are difficult for miniaturization for miniaturized electrochemical energy storage devices and electrochemical micro-sensor applications. Furthermore, conventional REs are problematic in applications using non-aqueous electrolytes due to (i) unwanted liquid junction potentials and (ii) the contamination of either RE or the electrolyte solution by undesirable species [3,4]. Quasi-reference electrodes (QRE), typically just a metal wire (e.g., Ag or Pt) are often used in non-aqueous solvents or electrolytes. Assuming there is no change in the bulk solution, the potential of QRE, although unknown, will maintain constant during the experiments. However, the actual potential of QRE vs. a true reference electrode must be calibrated under the same conditions. QRE is not suitable in experiments in which changes in the composition of the bulk solution can cause concomitant variations in the potential of QRE, such as bulk electrolysis or miniaturized electrochemical sensors [5,6].

One of the major research areas in electrochemistry is to develop miniaturized reference electrodes that can provide highly stable electrode potential with ease of fabrication at low cost for electrochemical micro-sensor development. Electrochemical micro-sensors are one of the most promising sensor platforms due to their high portability, low cost, simple structure, and fast response time. They are being developed for a broad range of applications, including ambient air-quality monitoring, occupational health and safety, biomedical diagnostics, industrial process control, and anti-terrorism [7,8]. A very promising approach is to develop all-solid-state REs, also referred to as liquid-junction free REs. The liquid-junction free REs are favored to realize the miniaturization of electrochemical systems, enabling their integration onto various substrates of different sizes and shapes and in some instances may be utilized in high-pressure and high-temperature conditions [9,10,11]. For instance, Presser and coworkers demonstrated that porous carbon can be used as a quasi-reference electrode in acidic and neutral aqueous solutions, exhibiting good stability and reliability [12]. Bohnke and coworkers developed and tested a ceramic-based RE (lithium lanthanum titanium oxide) in cyclic voltammetric experiments, during which comparable results to SCE electrodes were obtained. They also predicted that this material should be promising under high-pressure and high-temperature environments [13].

In addition to inorganic components, conductive polymers are shown as a versatile material for their application in the design as all-solid reference electrodes. Conductive polymers are a class of polymers that are able to conduct electricity. They are being explored as RE using different strategies. The first was to incorporate various inorganic particles (Hg/Hg_2_Cl_2_, Ag/AgCl or Fe(CN)_6_^3−/4−^anions) in conductive polymers to enhance their stability and adjust the ion transports. The second was to use bilayer or multi-layer conductive polymers as part of the RE system. The third was to directly use the conductive polymers as REs [14]. Mandler modified the glassy carbon electrode with polyethylenimine (PEI), a highly charged polymer that bound strongly with Fe(CN)_6_^3−/4−^anions. This modified glassy carbon was used as a relatively stable RE in organic solvents, but it requires incubation for several hours in acetonitrile before reaching a stable potential. In dimethylformamide (DMF), the stability was just several hours, and the measured potential drifted considerably after one day [15]. Bard proposed to use partially oxidized polypyrrole coated on metal (Pt or stainless steel) as a quasi-RE for use in acetonitrile, DMF, and aqueous solution but found its good stability was over just one day [16]. Ghilane used a platinum/poly (*N*-ferrocenylmethyl-*N*-allylimidazolium bromide) as a quasi-RE for non-aqueous (acetonitrile) and ionic liquid solutions ([EMI][TFSI]). The results showed better performance than the conventional metal wires, and it was stable for seven days. But it is still a quasi-RE, not a real RE [17]. Ferrocene or ferrocene derivative is often added into the non-aqueous electrochemical system when a quasi-reference electrode is used. The redox potential of the ferrocene is used for calibrating the RE electrode potential in many non-aqueous electrochemical systems [18].

Conductive polymers have inherent mechanical flexibility, printability, biocompatibility, and low cost [19]. They could have significant advantages over inorganic counterparts for developing miniaturized RE. Conductive polymers often refer to π-conjugated polymers [20,21,22]. Redox polymers are another separate class of polymers, which are composed of non-conjugated polymeric chains, with the redox-active moieties (ferrocene, viologen, quinone, TEMPO, etc.) as pendants. The conductivity of redox polymers is characterized by a “charge hopping” mechanism [23,24,25]. The π-conjugated polymers feature these organic moieties with the delocalized π electrons, which constitutes the necessary structural basis for their facile conductivity. Poly (acetylene), polyphenylene, poly (*p*-phenylene vinylene), polyaniline, polythiophene, and polypyrrole are several representative examples [26,27,28,29,30]. The fundamental and applied research of both π-conjugated and redox active polymers have been extensively investigated [31,32,33,34,35]. Generally, conductive polymers exhibit hybrid electronic–ionic transport properties, i.e., coupled electron/ion transfer, solvent transfer, and polymer reconfiguration. The transport of solvent or ion species within polymer films not only has a great impact on kinetics and thermodynamics of the redox behavior but also on the film surface structure [36,37]. The actual polymer structure that affects its electrochemical behavior strongly depends on the nature of the solvent and supporting electrolyte. The ideal conductive polymer-based reference electrode should have a simple Nernstian behavior of the polymer. The redox polymer-modified electrodes are often formed by electrochemical deposition of their polymer form since the monomer of the redox polymer cannot be easily polymerized on the substrate electrode. In contrast, monomer of the π-conjugated polymer can be easily oxidized to form thin films on various substrates through monomer electrochemical polymerization, allowing ease and controlled fabrication of conductive polymer thin films that enable novel electrochemical device architectures for next-generation bioelectronics, sensors, and energy conversion devices.

**Figure 1:**
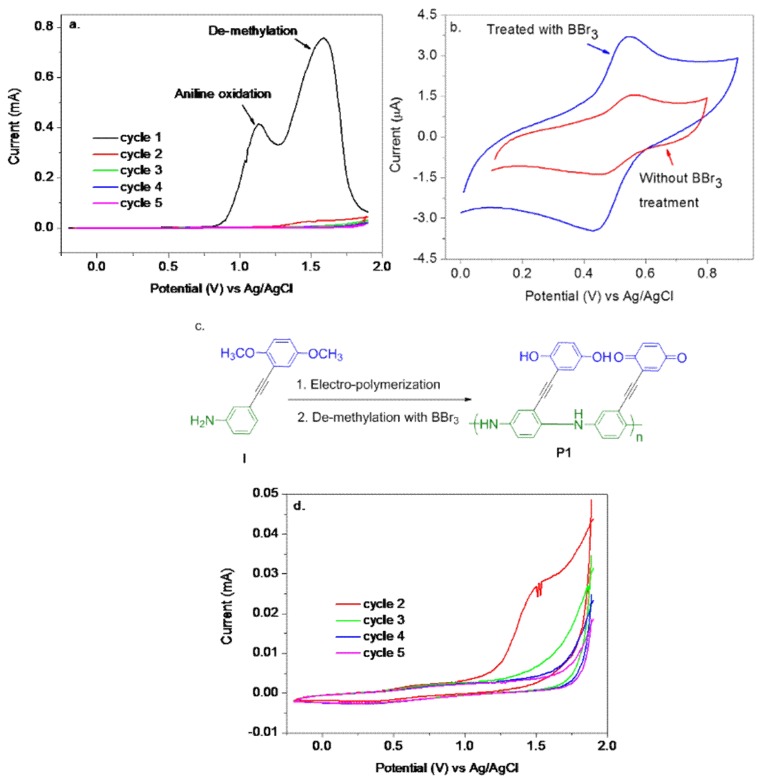
(**a**) Cyclic voltammetry of electro-polymerization of 50 mM Monomer **I** in anhydrous acetonitrile with 0.1 M tetrabutylammonium perchlorate (TBAP), potential window: −0.2 to 1.9 V, scan rate: 100 mV s^−1^. (**b**) Comparison of the electrochemical responses of **P1** in citric acid buffer (pH = 2.0) with or without the Lewis acid (BBr_3_) treatment, at a sweep rate of 25 mV s^−1^. (**c**) The overall reaction scheme of electro-synthesis and de-methylation. (**d**) Expanded cycle 2 to cycle 5 in (**a**).

In this work, a new type of redox conjugated polymer has been designed that combines the benefit of both redox and conjugated polymer as a solid-state RE. As quinone is a good redox label [38,39], it was selected as the redox pendant, and aniline was selected as a monomer to constitute the novel redox conjugated polymer. As shown in Figure 1c, an aniline monomer **I**, functionalized with *para*-dimethoxybenzene as latent “*Pro*-Redox” center was designed. This polymer design was supported by an early work in which polyaniline from mono-substituted anilines (2- or 3-methylaniline) has been found to have some catalytic effects towards hydroquinone–benzoquinone redox reaction [40].The two π-aromatic fragments were combined with a triple bond. One reason was from the viewpoint of easy synthesis. Another reason was that electronic communication could be minimized to ensure that the pendant group’s redox reaction does not affect the conductive polymer backbone [41]. Monomer **I** was synthesized through classical Sonogashira coupling and its electro-polymerization condition was explored, featuring a non-acid organic solvent-mediated method. The redox conjugated polymer has been electrochemically characterized and tested as an all-solid-state RE. The influence of the added quinone on the π-conjugated polyaniline backbone was investigated, gaining some critical insights into the collective electrochemical properties of this class of unique conductive materials. The potential applications of this novel poly (aniline quinone/hydroquinone) as an all-solid-state RE were tested in both aqueous and non-aqueous electrolytes. The stable electrode potential of the novel poly (aniline quinone/hydroquinone) suggested that it was a single phase in which the amounts of totally reduced and totally oxidized species could be maintained at a constant in various solvents and electrolytes. The aniline and quinone moiety in poly (aniline quinone/hydroquinone) render it to have both hydrophilic and hydrophobic compatibility with both aqueous and non-aqueous solvents so that its solvation and configuration can quickly reach a global equilibrium in both media.

## 2. Experimental Section

### 2.1. Chemicals

3-Ethynyl aniline (≥98%), acetonitrile (anhydrous, ≥99.8%), dichloroethane (≥99.0%), dichloromethane (≥99.5%), tetrabutylammonium perchlorate (TBAP for electrochemical analysis, ≥99.0%), boron tribromide (BBr_3_) solution (1.0 M) in methylene chloride, buffer solution pH 2.0 citric acid/hydrochloric acid/sodium chloride, ferrocenemethanol (FcMeOH, 97%), and 2-bromo-1,4-dimethoxybenzene (98%) were purchased from Sigma-Aldrich (Milwaukee, WI, USA). 

### 2.2. Synthesis of Monomer **I**

The Monomer **I** was synthesized using Sonogashira coupling between 3-ethynylaniline and 2-bromo-1, 4-dimethoxybenzene, two commercially-available starting materials. The synthesis scheme and NMR characterization are shown in Supplementary Materials. The procedure is as follows: after Pd(PPh_3_)_4_ (148 mg, 0.128 mmol, 5% eq.), CuI (49 mg, 0.256 mmol, 10% eq.), 3-ethynyl aniline (300 mg, 2.6 mmol), 2-bromo-1,4-dimethoxybenzene (612 mg, 2.8 mmol) were added to 12 mL THF/NEt_3_ (volume ratio: 3/1) under a nitrogen atmosphere, the mixture was heated to 60 °C and stirred overnight. After the solvents were removed via vacuum, the residue was extracted by ethyl acetate from water, dried over anhydrous sodium sulfate, concentrated and purified by silica gel (Rf = 0.35) chromatography (hexane/ethyl acetate = 6/1) to obtain 3-((2,5-dimethoxyphenyl)ethynyl) anilne. ^1^H NMR (400 MHz, CDCl_3_) δ 7.10 (t, *J* = 7.8 Hz, 1H), 7.01 (d, *J* = 2.4 Hz, 1H), 6.95 (d, *J* = 7.6 Hz, 1H), 6.88–6.84 (m, 1H), 6.83–6.79 (m, 2H), 6.63 (d, *J* = 12.0 Hz, 1H), 3.85 (s, 3H), 3.76 (s, 3H), 3.66 (br, 2H); ^13^C NMR (100 MHz, CDCl_3_) δ 154.7, 153.5, 146.4, 129.4, 124.3, 122.4, 118.3, 118.2, 115.9, 115.5, 113.4, 112.5, 94.6, 93.9, 56.8, 56.0. The ^1^H NMR, ^13^C NMR and mass spectra of the monomer are shown in Supplementary Materials.

### 2.3. Electrochemical Procedures

Glassy carbon working electrodes (diameter = 2 mm) were purchased from CH Instruments Inc. (Austin, TX, USA) and pretreated by polishing with 0.3 and 0.05 µm α-Al_2_O_3_ powder successively, followed by extensive rinsing with distilled water and ethanol and dried under nitrogen gas. The glass carbon electrode was further conditioned by scanning between −0.5 and 1.2 V in 0.1 M H_2_SO_4_ using cyclic voltammetry until stable current responses were obtained. During the electro-polymerization process, the glassy carbon electrode was used as the working electrode, the platinum wire (diameter = 0.5 mm) and the silver/silver chloride were used as the counter and reference electrodes respectively. The electrolytic solution consisted of 50 mM monomer in anhydrous acetonitrile with 0.1 M TBAP electrolyte. The electrochemical measurements were performed with the Gamry potentiostat (Gamry Instruments Inc., Warminster, PA, USA). The electrode was rinsed with anhydrous acetonitrile after electropolymerization and dried with nitrogen. The electrode was then soaked in 1.0 M BBr_3_ in dichloromethane overnightat room temperature under ambient atmosphere for complete removal of the methyl groups. 

## 3. Results and Discussion

### 3.1. Electrochemical Polymerization

The synthesis of polyaniline through electrochemical techniques has been established, utilizing several different approaches, ranging from potentiodynamic, potentiostatic to galvanostatic methods. Among them, the potentiodynamic technique allows the in-situ observance of the polymerization process [26,27] and was used here. Monomer **I** did not readily dissolve in the aqueous solution, owing to its strong hydrophobic nature, even in the presence of strong acids (HCl, H_2_SO_4_, HClO_4_), which are typically added for polyaniline polymerization. Several organic solvents (acetonitrile, dichloroethane, and dichloromethane) were screened in our attempt to electro-polymerize **I**. Figure 1a shows the cyclic voltammograms of this process in anhydrous acetonitrile. There was no obvious difference in the polymerization step among these three solvents. A purple-colored film of polymer was formed on the electrode surface following the electrochemical polymerization procedure.

Two distinct oxidation peaks were identified (Figure 1a). One was associated with oxidation of the aniline monomer at approximately 1.1 V, producing the cation radical for polyaniline chain growth. The other oxidation peak of approximately 1.6 V was due to the de-methylation of the parent *para*-dimethoxybenzene in the pendant group. The partial oxidation removal of the two methyl groups at ~1.6 V produced the redox-active quinone/hydroquinone from the initial redox inactive *para*-dimethoxybenzene form [27,30,42]. Thus, the term latent “*Pro*-Redox” center is used for reference it in Monomer **I**. Some unwanted side reactions could be avoided in synthesis if the reactive quinone or hydroquinone structures are directly utilized. Anhydrous acetonitrile was utilized to suppress the possible electro-dimerization of dimethoxybenzene, as observed by Buck et al. [43].

**Figure 2:**
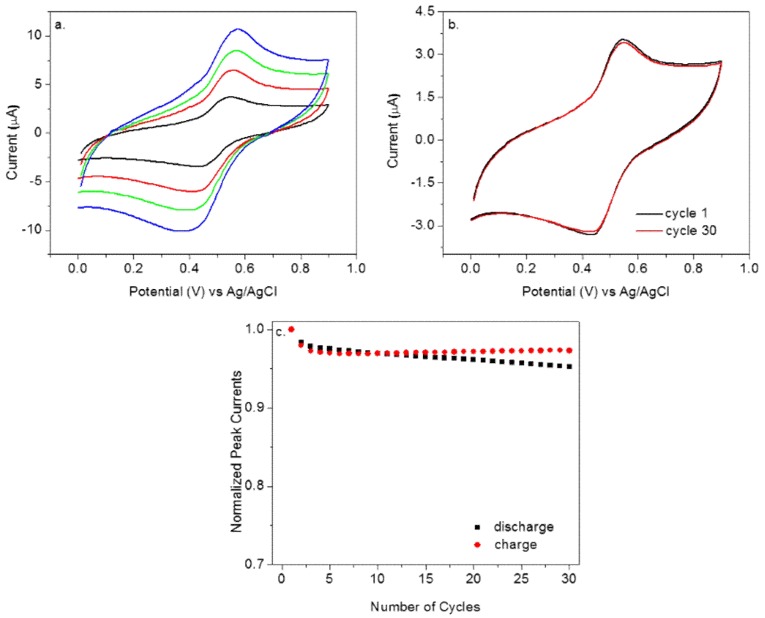
(**a**) Cyclic voltammetry of **P1** in citric acid buffer (pH = 2.0) at 25, 50, 75, and 100 mV s^−1^. (**b**) The electrochemical cycling of **P1** in citric acid buffer (pH = 2.0) at 25 mV s^−1^. (**c**) The normalized peak currents vs. the number of cycles, with normalized peak currents obtained by dividing each cycle’s peak currents over the first cycle’s peak currents.

BBr_3_, a strong Lewis acid, is used in chemical synthesis to remove methyl groups from methyl aryl ethers. A complex is formed between the electron-deficient boron and electron-rich oxygen, followed by bromomethane elimination to constitute the reaction mechanism (Figure S5) [44]. Thus, post-polymerization modification was performed to treat the pre-formed polymeric thin film with BBr_3_ to remove remaining methyl groups directly on the electrode surface. This two-step approach constituted an efficient sequence for **P1** formation, whose overall reaction scheme is displayed in Figure 1c. After BBr_3_ treatment, the electrochemical response of poly (aniline quinone/hydroquinone) **P1** was evaluated in the citric acid solution (Figure 1b). After the first oxidation sweep in electropolymerization, an obvious decrease in current was observed, which indicated the formed polymer was relatively poorly conducting and blocking the electrode from further reactions. BBr_3_ not only removed methyl groups but also could be beneficial to the improvement of polyaniline’s overall quality. It was clear that BBr_3_ was beneficial to enhance **P1**’s electrochemical performance by increasing the peak current intensity by ~140%. Previously Sjödin and coworkers did some elegant work in investigating the influences of different de-protection time using BBr_3_ on polypyrrole [45]. Herein, polyaniline was used as the backbone, which showed that **P1** still gave good electrochemical performance after an overnight treatment of BBr_3_ (Figure 1b and Figure 2a,b). This indicated that **P1** did not degrade or, at least, did not degrade too much. A possible reason might be that polyaniline was of the basic kind, so prolong interaction of BBr_3_ Lewis acid with basic polyaniline was tolerant during this process.

### 3.2. Electrochemical Properties of Poly(Aniline Quinone/Hydroquinone) **P1**

A redox peak pair observed for **P1**, Figure 1b, is the result of the redox conversion ofhydroquinone to benzoquinone with a formal potential E_Q_^0′^ of 0.49 V. The surface coverage was calculated to be 1.9 × 10^−8^ mol/cm^2^ according to Q/nFA (F is the Faraday constant, and A is the electrode area). Typically, polyaniline displayed two pairs of redox peaks, corresponding to leucoemeraldine/emeraldine and emeraldine/pernigraniline transitions respectively. Surprisingly, poly (aniline quinone/hydroquinone) **P1** did not show these two transitions. In other words, these characteristic polyaniline peaks have been “suppressed” by the quinone pendant. Only the quinone/hydroquinone conversion peaks were identified with an anodic and cathodic peak separation of 108 mV, which suggested relatively slow kinetics of the poorly conducting polymer. A possible explanation was the matched energy levels between polyaniline and quinone, which showed the quinone electron transfer on a conductive substrate electrochemically [46]. It is also likely that the protons in polyaniline itself can participate in the quinone redox conversion, being consistent with the observed electro-catalytic effects of poly (2-methylaniline) and poly (3-methylaniline [40]. Thus, **P1**’s reversible electron transfer redox behavior of pendant quinone/hydroquinone group is of great advantage to provide a stable reference electrode potential at electrode/solution interface since the solvent and ion transport, and the polymer reconfiguration processes are very fast. The redox reaction of **P1** can be written as follows:

Poly (aniline quinone) + 2e^−^ + 2H^+^ ⇔ Poly(aniline hydroquinone) (1)

(2) $${E=E}^{0}-\frac{0.059}{2}\log\frac{{{[H}_{2}Q]}_{\mathrm{polymer}}}{{\left[Q\right]}_{\mathrm{polymer}}}-0.059\mathrm{pH}$$

Polyaniline (base, N) + H^+^ ⇔ Polyaniline (acid, NH^+^) (3)

The polyaniline reaction in Equation (3) will affect the Nernst equation of **P1** in Equation (2) [47]. The electrochemical performance of **P1** was further characterized at different scan rates (Figure 2a). It was observed that the anodic peak potential shifted slightly in the positive direction with increasing scan rates while the cathodic peak potential shifted toward the negative direction. From the relationship between peak currents and scan rates, a surface-confined redox process was concluded. The electrochemical stability of **P1** was evaluated through multiple-cycle cyclic voltammetric testing (Figure 2b). It gave very stable responses under the testing conditions. The peak current retention was excellent, being about 95% for the continuous thirty cycles (Figure 2c), which opened up the possibility for its use as potential electric energy storage materials.

### 3.3. Poly (Aniline Quinone/Hydroquinone) as a Stable Reference Electrode

**Figure 3:**
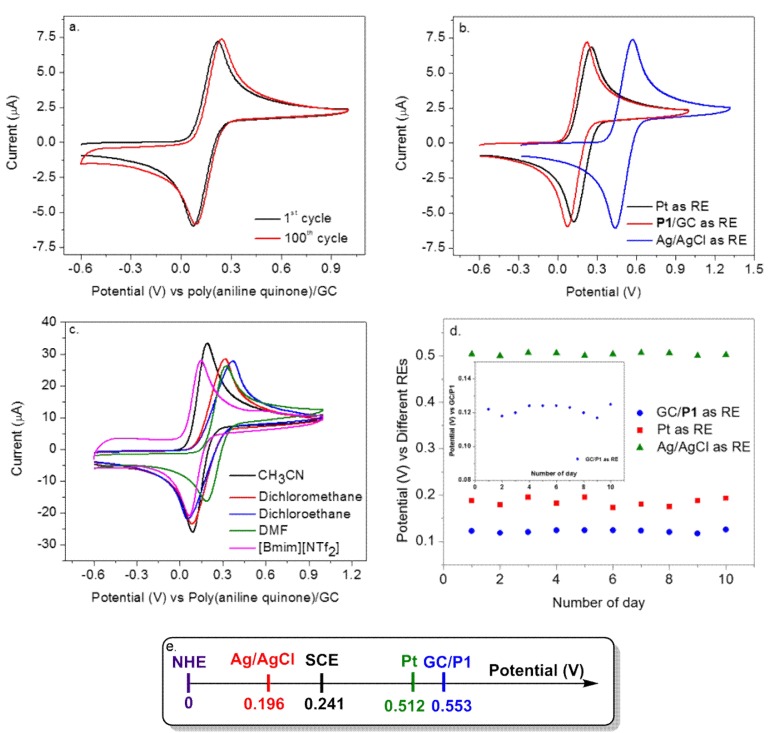
(**a**) The multi-cycle stability testing of **P1** as reference electrode (RE) in dichloromethane solution with 0.1 M TBAP electrolyte, with ferrocenemethanol (FcMeOH) used as redox probe. (**b**) Comparison of **P1** with Pt and Ag/AgCl reference electrodes, with FcMeOH used as redox probe. (**c**) The compatibility of **P1** as RE in different non-aqueous solutions, with FcMeOH used as redox probe. (**d**) The stability of GC/**P1**, Pt and Ag/AgCl as REs in dichloromethane solution with 0.1 M TBAP electrolyte, with FcMeOH used as redox probe and its average potentials plotted. (**e**) Scheme displaying the potential of GC/**P1** vs. the other common RE systems.

Unlike the aqueous systems, reference electrodes in non-aqueous solutions (organic solvents, ionic liquids, and molten salts) have not been well established [14]. The feasibility of this novel quinone-substituted polyaniline as a RE was investigated in several non-aqueous media. Cyclic voltammetry, which features a non-zero current condition, was recorded in the presence of 1 mM ferrocenemethanol (FcMeOH) as redox probe, using a gold electrode as the working electrode and **P1**-coated glassy carbon (GC/poly (aniline quinone/hydroquinone)) as RE in different media. The FcMeOH redox couple exhibited typically reversible peaks, and a small drift in potential was observed after 100 cycles of testing (Figure 3a), which showed the reliable stability of poly (aniline quinone/hydroquinone) **P1** as a RE. Figure 3b compared the performance of **P1** with two other common reference electrodes of Pt and Ag/AgCl systems. The potential of **P1** RE is 0.312 V vs. SCE or 0.553 V vs. NHE (Figure 3e). The half-wave potential, which was the average of FcMeOH anodic/cathodic redox potentials, was monitored vs. GC/**P1** RE for ten consecutive days (Figure 3d and Figure S6) and compared with those of Pt and Ag/AgCl as REs. A maximum drift of ~4.7 mV was observed, which showed **P1**’s excellent stability as a RE. It was kept in air without any special protection between these measurements. 

### 3.4. Various Factors to Influence Poly (Aniline Quinone/Hydroquinone) as a RE

**Figure 4:**
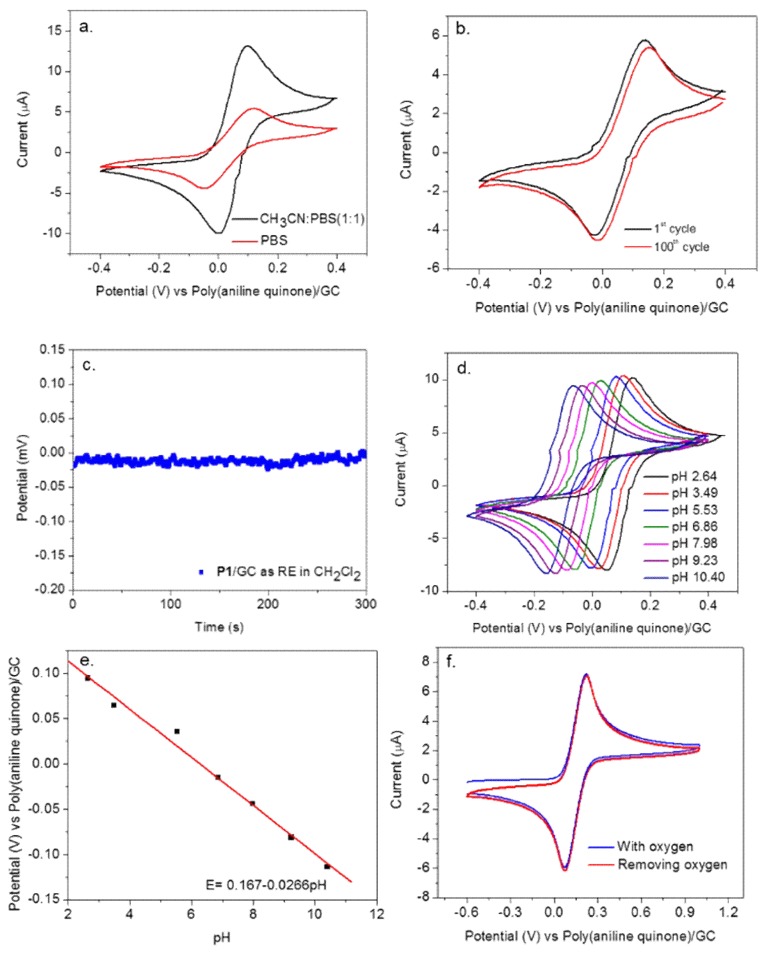
(**a**) Cyclic voltammetry of 1 mM FcMeOH, with **P1**-coated glassy carbon (GC/poly (aniline quinone)) as RE in PBS aqueous buffer and PBS/acetonitrile mixture. (**b**) Multi-cycle stability testing of **P1** as RE in PBS aqueous buffer; (**c**) Potentiometric measurement of P1 as RE under zero-current condition in dichloromethane solution with 0.1 M TBAP electrolyte, fluctuating within a range of 0.02 mV; (**d**) Cyclic voltammetry of 1 mM FcMeOH, with **P1** as RE in PBS with different pH values; (**e**) Average of anodic and cathodic peak potential vs. pH for 1 mM FcMeOH with **P1** as RE in PBS buffer of various pH; (**f**) Cyclic voltammetry of 1 mM FcMeOH, with **P1** as RE in dichloromethane with 0.1 M TBAP electrolyte with oxygen and without oxygen under nitrogen bubbling for ~20 min before measurement and the nitrogen atmosphere was kept above the solution during the testing.

**P1** was also tested in water (phosphate buffer saline (PBS) aqueous buffer) and water/organic solution (PBS/acetonitrile mixture). As shown in Figure 3c, this novel polymer was also compatible with various common organic solvents and [Bmim][NTf_2_] ionic liquid. The FcMeOH’s reversible pair was observed in different media and the peak potential of FcMeOH has been summarized in Table 1. The anodic peak potentials and anodic/cathodic potential average of FcMeOH were similar in dichloromethane (DCM), dichloroethane (DCE) and dimethylformamide (DMF) since these solvents were polar organic solvents. The anodic peak potentials of FcMeOH in PBS, CH_3_CN/PBS (1:1) and CH_3_CN were different from these polar organic solvents as they were either aqueous or miscible with water. This finding suggests that poly (aniline quinone/hydroquinone) shows an ideal Nernst behavior that is solvent independent under certain conditions. This is in great contrast to the traditional redox or conjugated polymer in which non-ideal behavior, the solvent dependence of all the equilibrium constants prevents meaningful comparison of electrochemical potential data obtained with a polymer film in different solvents. **P1** was also tested in water (PBS aqueous buffer) and water/organic solution (PBS/acetonitrile mixture) and was found to provide the stable and reproducible peak potentials in these systems as well (Figure 4a). The larger current observed in the water/organic mixture was due to the facile diffusion of FcMeOH within the organic media. Similar to the organic systems, **P1** behaved stably after 100 cycles of cyclic voltammetric testing in PBS aqueous buffer solution (Figure 4b). Moreover, a potentiometric measurement was conducted to show **P1**’s usefulness as a RE at the zero-current condition, within a negligible fluctuation range of 0.02 mV (Figure 4c). During the testing, gold electrode and platinum wire were used as the working and counter electrode respectively.

**P1**’s performance as a RE was also tested in PBS aqueous solutions with varying pH (Figure 4d). A ~25 mV shift was observed with one pH unit change, as expected due to the pH dependence of the quinone [48,49], but at fixed pH values, this RE showed stable potential values, validating its use as a RE. A linear relationship was established for the formal potential E^0′^ vs. pH, as shown in Figure 4e. A slope of 26.6 mV was obtained, which supports the proposed Nernst Equations (1)–(3). From Equation (2), quinone/hydroquinone potential should decrease with increasing pH, so the measured FcMeOH potential vs. quinone/hydroquinone should, thus, become larger at increasing pH. But interestingly, Figure 4e shows an opposite trend with a decrease of FcMeOH potential with increasing pH. As shown in Equation (3), protonation of polyaniline backbone can influence the potential of poly (aniline quinone/hydroquinone). Early work by Chiang and MacDiarmid showed that pKa of polyaniline is dependent on the pH and varies almost linearly between pH zero and seven [50]. Further work by H. Reiss explained that a proton adsorption isotherm exists in the emeraldine form of polyaniline-based on a strong repulsive interaction between protons on nearest-neighbor nitrogen atoms [51]. Thus, this unexpected observed pH dependence in Figure 4e is likely due to the complex interplay between multi-protic polymeric nature of polyaniline backbone and the quinone/hydroquinone with changing pH in poly (aniline quinone/hydroquinone). Furthermore, it has been found that oxygen did not influence **P1**’s performance. As shown in Figure 4f, **P1** was inert to the oxygen and stable in both oxygen and oxygen-free conditions. 

## 4. Conclusions

The combination of redox-active quinone and polyaniline was accomplished to give a novel redox conjugated polyaniline. The post-polymerization modification with boron tribromide furnished poly (aniline quinone/hydroquinone) **P1** with desired properties for its use as a solid-state RE. Electrochemical responses from polyaniline were suppressed by the quinone/hydroquinone pendants. It is expected that polyaniline is beneficial to provide the protons for the quinone/hydroquinone redox couple in aprotic organic solvents. **P1** was tested as a versatile all-solid-state RE in non-aqueous and aqueous media, including various organic solvents, an ionic liquid and PBS buffer at both non-zero current and zero-current conditions. The RE was stored in the air and there were no special protection conditions needed. During ten days of continuous testing, only a small drift of ~4.7 mV in potential was observed. Thus, the unique properties of poly (aniline quinone/hydroquinone) **P1** and its innovative polymerization methods described in this work will enable a new class of all-solid-state reference electrode that can be implemented in various electrochemical micro-devices.

## Figures and Tables

**Table 1:** Electrochemical parameters of 1 mM ferrocenemethanol (FcMeOH) with **P1** as reference electrode (RE) in various solvents. For DCM, DCE, DMF and CH_3_CN, tetrabutylammonium perchlorate (TBAP) was added as an electrolyte.

	DCM	DCE	DMF	[Bmim][NTf_2_]	CH_3_CN	PBS	CH_3_CN:PBS (1:1)
E_anodic_	0.312	0.373	0.319	0.143	0.189	0.119	0.0964
E_cathodic_	0.0865	0.0533	0.186	0.0663	0.0898	−0.0447	0.00137
ΔE	0.225	0.320	0.133	0.0767	0.0992	0.164	0.0950
Average	0.199	0.213	0.252	0.105	0.139	0.0371	0.0489

DCM: dichloromethane; DCE: dichloroethane; DMF: dimethylformamide; [Bmim][NTf_2_]: 1-Butyl-3-methylimidazolium bis(trifluoromethylsulfonyl)imide; PBS: phosphate buffer saline.

## Supplementary Materials

The following are available online at http://www.mdpi.com/2073-4360/10/11/1191/s1, Figure S1: the synthesis scheme, Figure S2: ^1^H NMR spectrum, Figure S3: ^13^C NMR spectrum, Figure S4: Mass spectrum, Figure S5: Mechanism of aniline oxidation and demethylation, Figure S6: Stability of GC/P1 as RE in different solvents.
