# Supplementary material for: A Redox Conjugated Polymer-Based All-Solid-State Reference Electrode
Source: Polymers (Basel). 2018 Oct 25;10(11):1191. doi: 10.3390/polym10111191 (PMC6290589; doi:10.3390/polym10111191)
Supplement: Supplementary file 1 [file polymers-10-01191-s001.pdf]

A redox conjugated polymer-based all-solid-state reference electrode

Ke Qu[1], Mingxi Fang[2], Shuwei Zhang[2], Haiying Liu[2], Xiangqun Zeng*[1]

1. Department of Chemistry, Oakland University, Rochester, Michigan 48309, United States
2. Department of Chemistry, Michigan Technological University, Houghton, Michigan 49931, United States

Supplementary Materials

1. The synthesis scheme and ^{1}H, ^{13}C NMR spectra of the synthesized compounds.

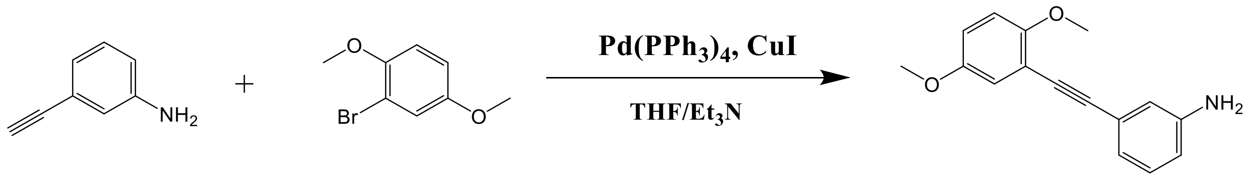

Figure S1. The synthesis scheme

To a N_2 protected 50ml round-bottom flask, $Pd(PPh_3)_4$ (148 mg, 0.128 mmol, 5% eq), CuI (49 mg, 0.256 mmol, 10% eq), 3-Ethynyl aniline (300 mg, 2.56 mmol), 2-bromo-1,4-dimethoxybenzene (612 mg, 2.8 mmol) were dissolved in 12 mL THF/Et_3N(3/1), then the reaction was heated to 60°C and stirred overnight. The solvents were removed via vacuum, the residue was extracted by ethyl acetate from water, dried over anhydrous sodium sulfate, finally purified by silica gel (R_f=0.35) chromatography (hexane/EA=6/1) to obtain the targeted compound. ^{1}H NMR (400 MHz, $CDCl_3$) δ 7.10 (t, *J* = 7.8 Hz, 1H),7.01 (d, *J* = 2.4 Hz, 1H),6.95 (d,*J* = 7.6 Hz, 1H), 6.88-6.84 (m, 1H), 6.83-6.79(m,2H), 6.63 (d, *J* = 12.0 Hz, 1H),3.85 (s,3H), 3.76 (s, 3H), 3.66 (br, 2H); ^{13}C NMR (100 MHz, $CDCl_3$) δ 154.7, 153.5, 146.4, 129.4, 124.3, 122.4, 118.3, 118.2, 115.9, 115.5, 113.4, 112.5, 94.6, 93.9,56.8, 56.0.

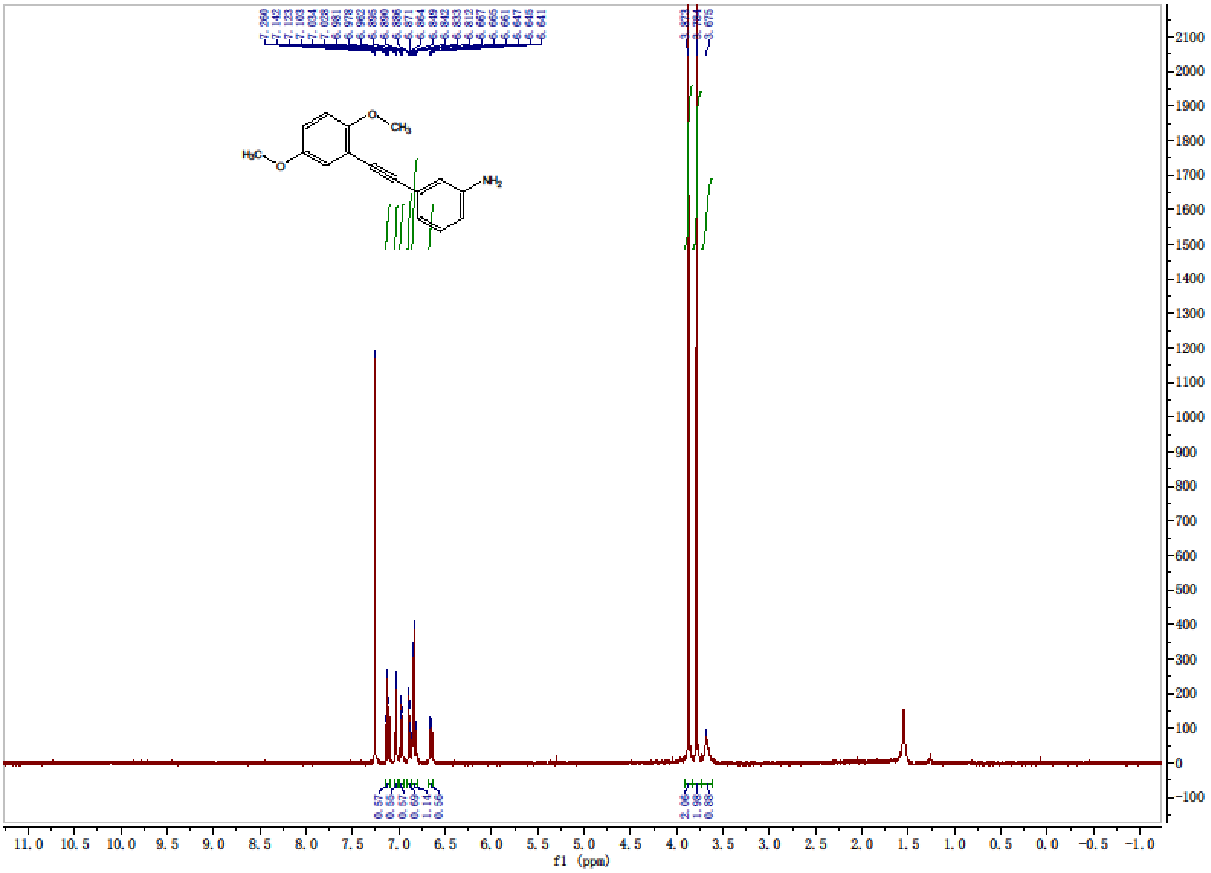

Figure S2. ^{1}H NMR spectrum

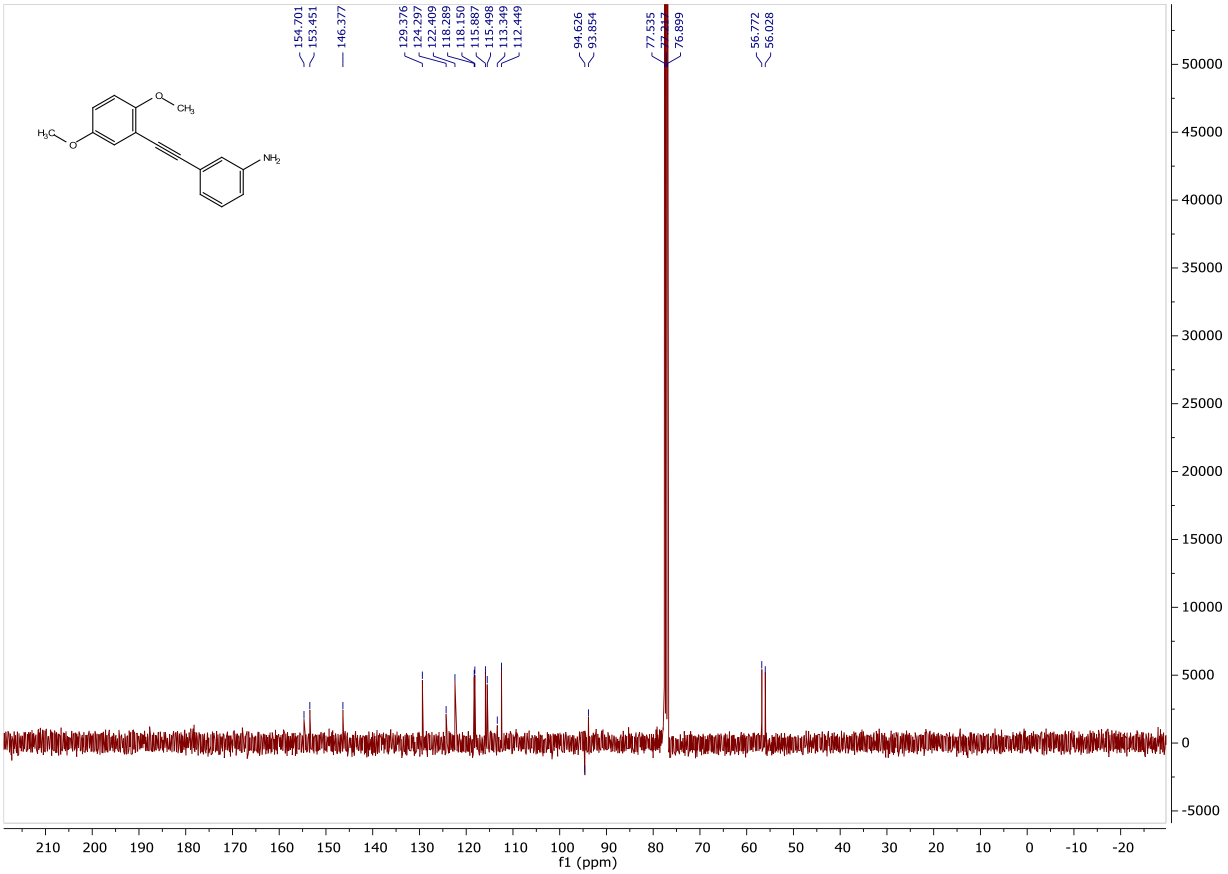

Figure S3. ^{13}C NMR spectrum

FTL-14

[Elemental Composition]

Page: 1

Data : 16Feb02_E02-002

Date : 02-Feb-2016 16:15

Sample: FTL-14

Note : 70eV

Inlet : Direct

Ion Mode : EI+

RT : 0.29 min

Scan#: (4,5)

Elements : C 400/0, H 800/0, O 2/2, N 1/1

Mass Tolerance : 10mmu

Unsaturation (U.S.) : -0.5 - 10000.0

Observed m/z	Int%	Err[ppm / mmu]	U.S.	Composition
253.1098	100.0	-1.7 / -0.4	10.0	C 16 H 15 O 2 N = 253.1103

[Mass Spectrum]

Data : 16Feb02_E02-002

Date : 02-Feb-2016 16:15

Sample: FTL-14

Note : 70eV

Inlet : Direct

Ion Mode : EI+

Spectrum Type : Normal Ion [EF-Linear]

RT : 0.29 min

Scan# : (4,5)

BP : m/z 253.1098

Int. : 26.33

Output m/z range : 200.0000 to 300.0000

Cut Level : 0.00 %

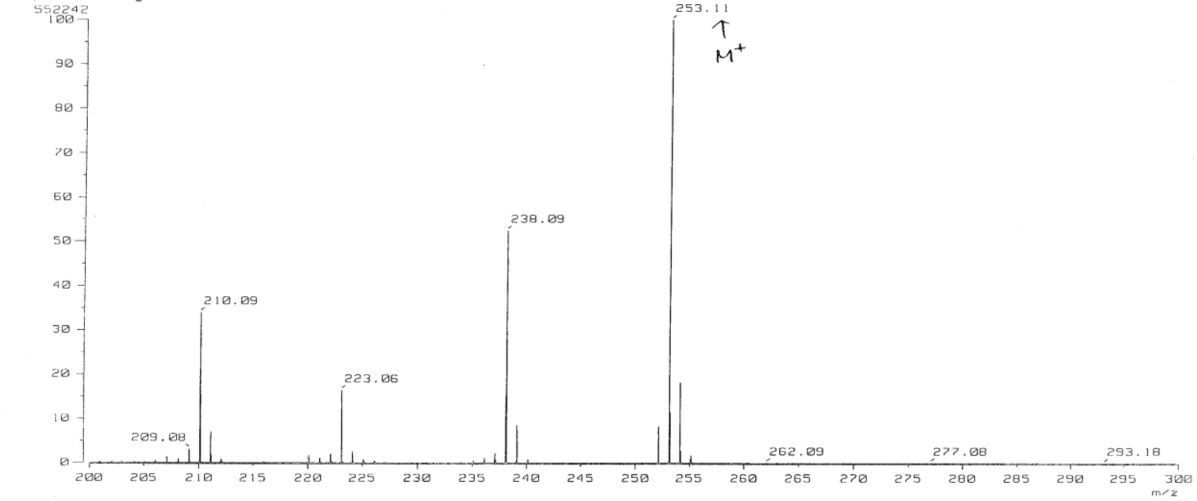

Figure S4. Mass spectrum

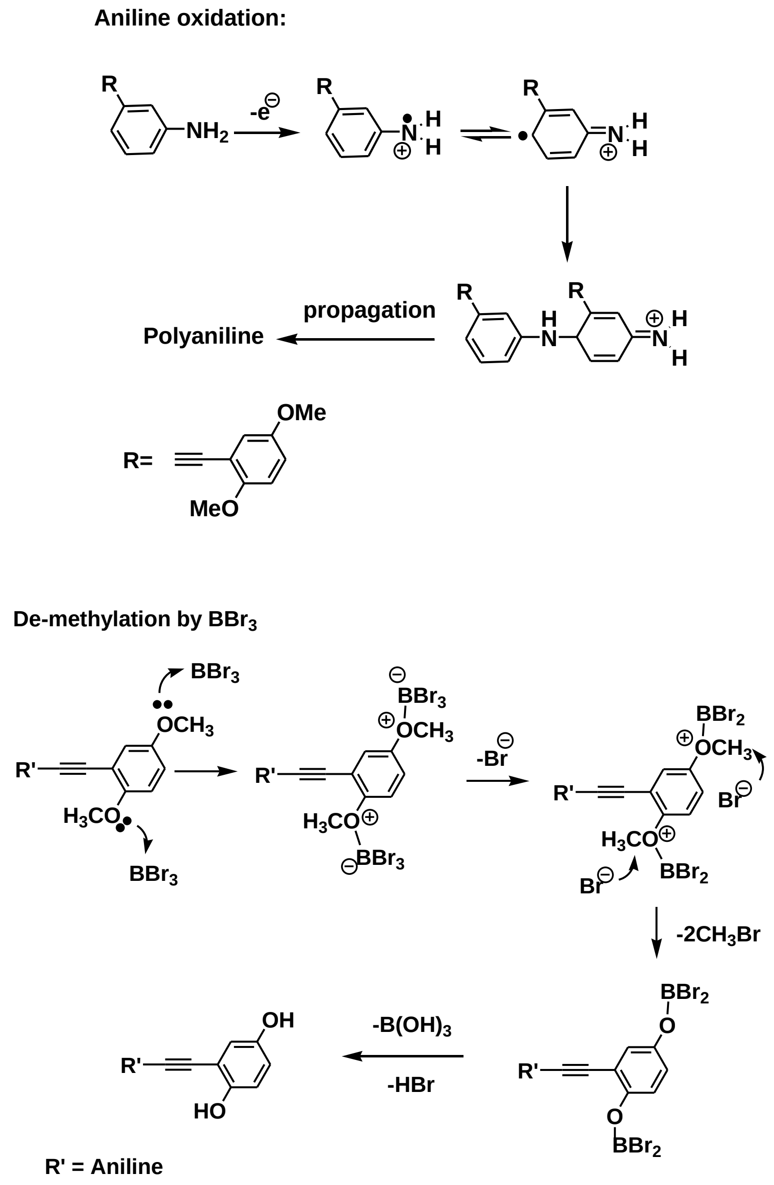

Figure S5. Mechanism of Aniline oxidation and de-methylation by BBr_3

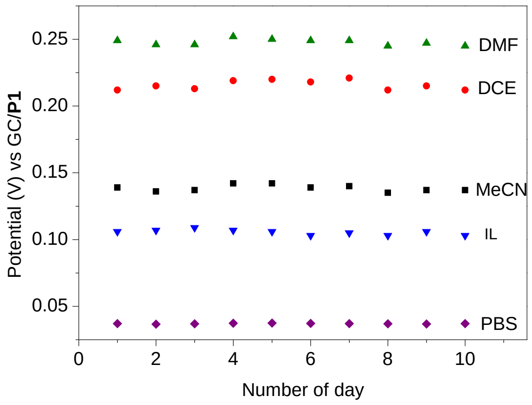

Figure S6. Stability of GC/**P1** as RE in DMF, DCE, MeCN, [Bmim][NTf_2] ionic liquid and PBS, with FcMeOH as redox probe and its average potentials plotted.
